# Global Avian Influenza Surveillance in Wild Birds: A Strategy to Capture Viral Diversity

**DOI:** 10.3201/eid2104.141415

**Published:** 2015-04

**Authors:** Catherine C. Machalaba, Sarah E. Elwood, Simona Forcella, Kristine M. Smith, Keith Hamilton, Karim B. Jebara, David E. Swayne, Richard J. Webby, Elizabeth Mumford, Jonna A.K. Mazet, Nicolas Gaidet, Peter Daszak, William B. Karesh

**Affiliations:** EcoHealth Alliance, New York, New York, USA (C.C. Machalaba, S.E. Elwood, K.M. Smith, P. Daszak, W.B. Karesh);; World Organisation for Animal Health, Paris, France (S. Forcella, K. Hamilton, K.B. Jebara, W.B. Karesh);; Ministry of Environment and Water, Dubai, United Arab Emirates (K.B. Jebara);; US Department of Agriculture, Athens, Georgia, USA (D.E. Swayne);; St. Jude Children’s Research Hospital, Memphis, Tennessee, USA (R.J. Webby);; World Health Organization, Geneva, Switzerland (E. Mumford); University of California, Davis, California, USA (J.A.K. Mazet);; French Agricultural Research Centre for International Development, Montpellier, France (N. Gaidet);; International Union for the Conservation of Nature Species Survival Commission, New York (W.B. Karesh)

**Keywords:** influenza, influenza virus, viruses, wild birds, global avian influenza surveillance, viral diversity, epidemiologic monitoring, molecular evolution, genetic variation, genomic library, animal diseases, zoonoses, genetic databases, disease reservoirs, Organisation for Animal Health, OIE, One Health

## Abstract

Wild birds play a major role in the evolution, maintenance, and spread of avian influenza viruses. However, surveillance for these viruses in wild birds is sporadic, geographically biased, and often limited to the last outbreak virus. To identify opportunities to optimize wild bird surveillance for understanding viral diversity, we reviewed responses to a World Organisation for Animal Health–administered survey, government reports to this organization, articles on Web of Knowledge, and the Influenza Research Database. At least 119 countries conducted avian influenza virus surveillance in wild birds during 2008–2013, but coordination and standardization was lacking among surveillance efforts, and most focused on limited subsets of influenza viruses. Given high financial and public health burdens of recent avian influenza outbreaks, we call for sustained, cost-effective investments in locations with high avian influenza diversity in wild birds and efforts to promote standardized sampling, testing, and reporting methods, including full-genome sequencing and sharing of isolates with the scientific community.

Avian influenza is a global threat to food animal production and distribution systems, as well as human health. However, sustained, comprehensive, and coordinated global efforts to monitor the continually changing genetic diversity of avian influenza viruses circulating in nature are lacking (*1*,*2*). Two avian influenza viruses are current pandemic threats: highly pathogenic avian influenza A(H5N1) virus, which has spilled over repeatedly to humans since its first report in 1996; and novel avian influenza A(H7N9) virus, first detected in March 2013, which has caused serious human infections across China. Wild birds played a role in the evolution of influenza A(H7N9) virus (*3*) and might have contributed to the spread of this virus to parts of Asia, Europe, and Africa after a 2005 outbreak in birds at Qinghai Lake in China (*4*,*5*).

Most recently, wild birds have been considered as having a role in the unexpected appearance and spread of influenza A(H5N8) virus in Europe in November 2014 and in North America in December 2014; genetically similar lineages have also been found in South Korea and Japan (*6*). Concern about highly pathogenic avian influenza viruses has generated sporadic attention and investments paired to specific subtypes. For example, alarm about influenza A(H5N1) virus resulted in short-term spending of hundreds of millions of dollars for wild bird–related research globally. Although interest and funding has since waned, threats from avian influenza viruses remain, and H5N1 and H7N9 subtype viruses continue to cause human infections and deaths.

Wild birds are natural reservoirs for avian influenza virus (*4*,*7*), host a wide diversity of subtypes, and provide a dynamic population for viral evolution and transmission to domestic flocks and mammals. Most hemagglutinin (HA) and neuraminidase (NA) subtypes have been detected in wild birds, although some infrequently (*8*,*9*). Highly pathogenic avian influenza viruses of poultry are defined by the World Organisation for Animal Health (OIE) as having an intravenous pathogenicity index >1.2 in 6-week-old chickens and causing ≥75% deaths in 4 to 8-week-old chickens infected intravenously, or H5 or H7 virus isolates with a characteristic molecular sequence at the HA cleavage site (for full definition, see the OIE Terrestrial Animal Health Code [*10*]). Although these viruses are rarely detected in wild birds, they have been found in diverse wild bird species from disparate locations and can be potentially transmitted along transcontinental flyways (*5*).

Avian influenza rarely causes widespread deaths in wild birds, and influenza caused by H5N1 subtype virus represents the first major clinical avian influenza virus–associated disease recognized in wild birds since the outbreak of influenza caused by H5N3 subtype virus in South Africa in 1961 in common terns (*Sterna hirundo*) (*7*,*11*). Thus, limiting surveillance for avian influenza virus to only deaths of wild birds provides little insight into the diversity of avian influenza virus genotypes circulating globally or risk for future outbreaks in poultry or humans.

Programs implemented during sporadic periods of concern (e.g., the Global Avian Influenza Network for Surveillance of Wild Birds after influenza outbreaks caused by H5N1 subtype viruses in 2005 [*12*]) have provided useful data on avian influenza virus diversity for a limited number of isolates but have missed the opportunity to document how diversity changes over time (*1*). The situation for wild bird surveillance parallels similar constraints for surveillance of influenzas circulating in swine, as highlighted in a recent review (*13*).

While focusing on wild birds, we recognize the benefits of globally coordinated surveillance systems for all influenza viruses with the aims of multitype detection, whole-genome sequencing, sharing of virus isolates, and analysis of epidemiologic data to highlight changes in circulating virus subtype prevalence. In contrast to our current system, which largely emphasizes reacting to new avian influenza viruses once they are detected in poultry, more upstream tracking of this information could potentially provide a critical early warning system or at least provide a sense of the likely evolution and movement of these viruses so that more proactive action can be taken.

Wild bird surveillance information could directly benefit human and animal health through understanding of how avian influenza virus genes flow into poultry, swine, equine, and human influenza viruses and could provide a basis for strategies that reduce their risk for introduction into agricultural species and humans. The documented human case of influenza in Taiwan in 2013 caused by an H6N1 subtype virus (*14*) points to the potential value of broader avian influenza virus surveillance in other species because this subtype has rarely been included in influenza surveillance systems.

In recognition of the potential benefits of surveillance, the OIE–Food and Agriculture Organization global network of expertise on animal influenza (OFFLU) established a working group on wildlife influenzas in October 2014; this group has highlighted the need for wild bird surveillance to understand circulation dynamics of avian influenza virus and recommends full-genome sequencing (*15*). In April 2014, the Strategic Alliances for the Coordination of Research on the Major Infectious Diseases of Animals and Zoonoses identified avian influenza surveillance in wild birds as a top priority for collaboration as part of a 10-year strategic research agenda (*16*). We review current surveillance efforts and provide recommendations toward establishing an effective surveillance program for avian influenza in wild birds.

## Assessing the Current State of Wild Bird Surveillance

A global look at multiple major data sources on wild bird surveillance for avian influenza viruses by country has not been previously reported. An excellent review by Hoye et al. (*1*) analyzed 191 literature reports of wild bird surveillance initiated during 1961–2007 and identified needs for a coordinated scientific approach, including refined sampling and screening strategies. Our analysis incorporates additional surveillance and viral diversity data sources available at a global level and assesses current/recent efforts to determine capacity and actions needed for an effective avian influenza virus tracking system in wild birds (Technical Appendix). Other sources of information (e.g., in local languages) might provide further useful information for future analyses.

## OIE Member Survey

 Forty-six (25.8%) of 178 OIE member countries responded to an influenza surveillance survey (Technical Appendix). Eleven (23.9%) of 46 responding countries reported active (live birds) and passive (dead birds) surveillance activities (whether collected specifically for the study or by other means [e.g., sampling of hunter collections]); 14 (30.4%) reported active surveillance only; 14 (30.4%) conducted passive surveillance only; and the remaining 7 (15.2%) reported conducting no surveillance activity. Several of these countries cited a lack of funding as the impediment. Of 39 countries that reported surveillance (Technical Appendix), 23 (58.9%) specified testing for virus subtypes deemed to be a higher pathogenic risk to poultry and humans (H5, H7, and all highly pathogenic avian influenza viruses identified) and 9 (23.0%) reported that they tested to determine all subtypes found. Only 11 subtype combinations were reported through the OIE survey from 9 countries during January 2012–March 2013.

## OIE–World Animal Health Information Data Interface

A total of 116 countries submitted reports during the period examined (2008–2012) (*17*). Reports indicated that 82 of those countries conducted some form of avian influenza viral surveillance in wild birds (Technical Appendix). One country reported highly pathogenic avian influenza virus (H5N1 subtype), 6 reported low pathogenicity avian influenza during 2008–2012 (including 10 HA segments and 39 subtype combinations), and the remainder reported no viruses. The other countries did not report detection. Of the 682 entries of highly pathogenic and low pathogenicity viruses detected in wild birds from the 37 countries reporting detection or suspicion of avian influenza, 244 entries were at the serotype (HA type or subtype combination) level of information.

## Influenza Research Database

A total of 39 countries (Technical Appendix) reported wild bird surveillance. This surveillance, which was based on species name, was conducted during January 2008–June 2013.

## Web of Knowledge

At least 54 countries reported wild bird surveillance during 2008–2013. This surveillance was reported mainly through peer-reviewed literature.

We found that ≥119 countries conducted and reported some form of avian influenza virus surveillance in wild birds during January 2008–January 2013 (Technical Appendix). We identified 3 trends in surveillance efforts and their implications for comparability of data, as well as opportunities for refining surveillance strategies to capture diversity of avian influenza viruses.

The first trend was sampling method (active versus passive surveillance; number of birds and species sampled; types of samples collected as cloacal, oropharyngeal/tracheal, fecal, blood, or tissue samples; sampling site characterization; frequency and seasonality of sampling) differs widely across surveillance programs. This finding is consistent with analysis of surveillance efforts of Hoye et al (*1*) conducted during 1961–2007 and suggested that unstandardized sampling approaches remain a chronic challenge for a global avian influenza virus surveillance system. Sampling methods, although not necessarily a limiting factor in identifying the wide range of these viruses circulating in nature, pose difficulties in discerning the usefulness of a report and comparing fluctuations in subtype virus prevalence between years or areas.

The second trend was that testing and virus characterization protocols vary widely. Some countries or programs screen for influenza A virus, some selectively screen for H5 and H7 virus subtypes, and some screen only for HA and NA virus subtypes, but not subtype virus combinations. Although we acknowledge resource and capacity limitations for analysis, the high level of effort required to capture or sample wild birds argues for investment to test for virus subtypes in addition to those believed to be currently highly pathogenic, to sequence as many as economically feasible, and to share samples and genomic data widely. Sequence data are needed to effectively track viral diversity, spread, and evolution.

The third trend was that critical data are deficient in most reporting systems. Measures of sampling effort are often absent. None of the data sources reviewed confirmed that a country did not conduct surveillance, which confounded analyses of negative findings. Approximate location or date of positive and negative findings is often missing, which hinders spatiotemporal risk analyses. Because positive findings are commonly reported without denominator data, especially for H5 and H7 subtype viruses, relative risk for spillover is difficult to assess. Only the Influenza Research Database (*18*) enables aggregation of full genetic sequences. Finally, analysis of which types of samples yielded more positive results (e.g., cloacal swab versus fecal specimens) is not possible from these data, which limits their value in informing future sampling strategies.

We recognize that studies have had varied aims that contribute to differences in surveillance methods. However, the lack of standardized, sustained, and targeted surveillance prevents effective tracking of avian influenza virus diversity over time in the major reservoir for this group of viruses. Opportunities for systematic avian influenza virus surveillance could have been readily implemented through existing programs. For example, regional guidance through the European Union (EU) directives on wild bird surveys has promoted some level of comparability of data through a required uniform reporting format, which led to extensive data sharing. In 2006, EU member states reported having tested 120,706 birds (active and passive surveillance) in ≥330 species of 22 orders (*19*). Unfortunately, samples were not screened beyond influenza A virus (or, in some cases, H5 or H7 subtype viruses) under the EU directive. Future regional coordinated efforts could begin to increase avian influenza virus tracking even if only requiring full genome sequencing on a subset of samples. Ongoing systematic surveillance can inform avian influenza virus ecology research, going beyond pattern descriptions or experimental conditions by generating long-term data to address complex processes around dynamics of host immunity or viral diversity (*2*).

## Capturing Viral Diversity

Improved understanding of avian influenza virus need not involve a monumental global effort but requires a shift in screening practices to move beyond emphasis on highly pathogenic avian influenza viruses. Where available, genomic sequencing for detection of avian influenza virus may provide more robust subtype findings.

A recent study detected by sequencing different HA subtype viruses that had not been detected by antigenic subtyping approaches (*20*). However, we call for a phase change in surveillance programs, which includes additional measures to track avian influenza virus diversity beyond subtype, at least in a subset of samples. Capturing information on all 8 avian influenza virus gene segments by routinely sequencing wild bird isolates would enhance understanding of avian influenza virus dynamics even more. For example, influenza A(H7N9) virus that caused respiratory disease in humans evolved from genetic contributions of ≥4 gene segment origins, including sources from wild birds (*3*). These contributions exemplify complex major genetic evolution around antigenic drift and gene reassortment, which subtype characterization alone does not capture.

The 6 less well-studied influenza virus gene segments may confer major determinants of infectivity, pathogenicity, transmissibility, and host species susceptibility (e.g., the role of the basic polymerase 2 gene segment in host range and virulence) (*9*). Tracking gene segments may be especially useful for wild birds, given the high reassortment rate in their avian influenza virus genome sequences (*21*) and the unknown potential for transient genomes to become stable and pose risks for endemic infections or transmissibility to or pathogenicity in humans. As a long-term goal, surveillance should be refined to maximize understanding of transmission factors (e.g., host receptivity and susceptibility, host dispersal) and ecologic factors (e.g., climate-dependent viral persistence, migration timing and range) that drive avian influenza virus prevalence in wild birds (*22*) and enable spillover, emergence, and maintenance (*1*).

Coordinating bodies, such as OFFLU, have an opportunity to develop specific standards for avian influenza virus diversity-oriented surveillance programs to ensure a clear strategy forward for the scientific community and its funders. Similarly, funders have a key role in driving efforts to track avian influenza virus diversity but will have to embrace sustained screening efforts for highly pathogenic and low pathogenicity avian influenza viruses and promote the value of increased sharing of negative findings and require full-genome sequencing of avian influenza viruses, even if required for only a subset of samples.

## Reporting Systems

Despite benefits of international sharing of avian influenza viruses, regardless of their pathogenicity, there is no standardized and comprehensive reporting requirement beyond highly pathogenic avian influenza virus and H5 and H7 subtypes of low pathogenicity avian influenza viruses, nor adequate reporting incentives. Scientific journals and online databases enable sharing of information from research projects, as well as from official government surveillance, but these sources inherently underreport negative findings, and the lack of standardization among studies reduces value to authorities responsible for prevention and control. Online databases, such as the Influenza Research Database and GenBank, provide forums for detailed and consistent reporting, including sequence data.

We urge avian influenza virus surveillance funders to require reporting of results to the Influenza Research Database to drive sharing of metadata and genetic sequence information. This sharing would expand the utility of local- and national-level data to feed into global data analysis. The limited current comparability of data can inform database directors on the need to harmonize data deposition requirements, improve interoperability, and encourage full reporting of negative and positive results to optimize tracking potential for avian influenza viruses. Ongoing discussions between coordinating bodies, such as OFFLU, database designers, and funders, might help improve efficiency and refinement of reporting systems and also ensure resource alignment across high-priority surveillance and reporting. Overall, a coordinated surveillance system should capitalize on global sharing tools to promote access to information that will support rapid detection of these virus and ongoing analysis.

## Targeted Country Participation

A coordinated, annual surveillance system with a global perspective does not necessarily require participation from every country. Rather, resource allocation could be prioritized to provide sustained surveillance in a few targeted locations and in specific seasons that maximize information on viral diversity relevant to potential spread (e.g., high-risk species, species interfaces, major staging and migration stopover sites, and reassortment hotspots [*23*]). Recent analysis of avian influenza virus subtype diversity and richness suggests that 75% of HA and NA subtype diversity in wild birds could be captured through targeted surveillance efforts in the Northern Hemisphere over a 4-year period (*24*).

In addition, findings recently reported from the largest avian influenza surveillance program in wild birds ever implemented (a study by the US Department of Agriculture and Department of Interior in collaboration with authorities in Canada and Mexico) suggested that hotspots of avian influenza virus in wild birds were primarily located in the northern latitudes of the United States (*25*). These findings suggest that sites in the Northern Hemisphere are high-yield starting locations for viral diversity, especially because we found that countries considered as major sources of avian influenza virus diversity (e.g., Canada, China, Germany, Mongolia, Norway, Russia, the United Kingdom, and the United States) have reported surveillance data since 2008, but to different data portals (Figure; Technical Appendix).

**Figure:**
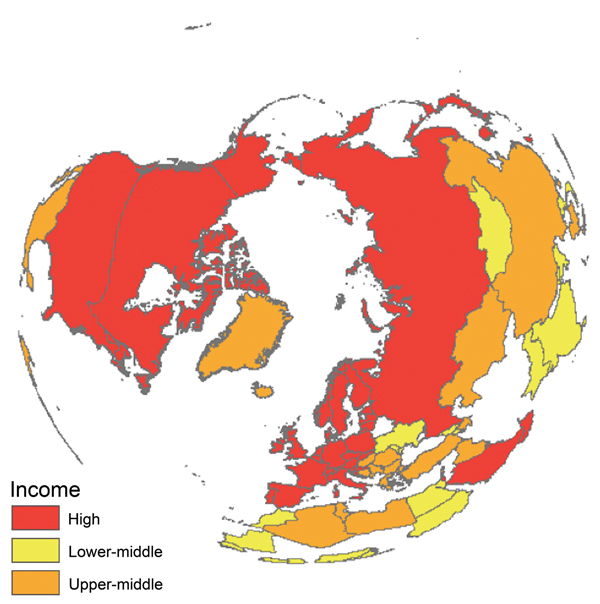
Feasibility of coordinating and improving avian influenza surveillance in wild birds where viral diversity is highest. Countries in red, orange, and yellow currently self-report some type of avian influenza surveillance in wild birds (For a country list, see online Technical Appendix, http://wwwnc.cdc.gov/EID/article/21/4/14-1415-Techapp1.pdf). Country income levels, based on gross domestic product, further suggest financial capacity to contribute to a coordinated surveillance system. The polar view emphasizes where most avian influenza viral diversity is circulating.

Coordinating extant programs in areas with major roles in avian influenza virus diversity would be a cost-effective first measure. Although we propose an initial focus on the Northern Hemisphere to leverage current investments and target surveillance on the basis of prior avian influenza study findings reported above (*24*,*25*), we do not intend to undermine the role of efforts elsewhere. We especially acknowledge that bias in surveillance effort has limited current knowledge on avian influenza virus diversity in the Southern Hemisphere (*4*). Surveillance in the Southern Hemisphere, where resources are available, provides highly useful information on exchange in the avian influenza virus genetic pool through bird migration (migration–shedding dynamics may enable avian influenza virus dispersal over extensive distances [*5*]), poultry trade, and maintenance of some specific phylogenetic lineages of these viruses.

## Study Limitations

Our analysis was intended to provide a snapshot of recent avian influenza virus surveillance effort in wild birds to explore how existing infrastructure could be optimized to capture viral diversity. There are limitations of our study, including information missed through our data compilation methods. Only a subset of OIE member countries responded to the survey, and some responses were incomplete or unclear, which was potentially caused by instructions or language barriers. In addition, because only highly pathogenic influenza viruses or influenza A H5 or H7 subtype viruses are required for reporting to OIE, data for low pathogenicity avian influenza viruses were reported voluntarily by countries. Thus, reports to OIE cannot be assumed to be comprehensive.

We targeted information specific for wild birds, but it was not always possible from the data sources reviewed to determine whether animals were truly free-ranging or captive (e.g., data from the World Animal Health Information Data Interface [WAHID] was specific for wild species but did not distinguish by setting, which might show different dynamics for avian influenza viruses). Articles reporting surveillance from the Web of Knowledge were reviewed for specific parameters (e.g., time frame, wild bird species), but this method excluded papers not explicitly reporting this information. Additional relevant papers were likely available through other databases.

Non-reporting does not indicate lack of surveillance and likely increases the number of countries actually conducting surveillance. We also recognize that reports of surveillance are not always verified for accuracy, and surveillance at one time point might not indicate current capacity. We included sampling effort reported that occurred during 2008–2013, but not all data sources spanned that time frame (e.g., the OIE survey only reflected 1 year of activities). Last, because in some cases it was not possible to ascertain the lead institution organizing surveillance efforts in a given country (government versus in-country research organizations or outside institutions), this information was not compiled. We acknowledge that outside research might not be indicative of true in-country capacity and might have different implications for reporting. Despite several limitations, our findings suggest that investments have been recently made for surveillance in most countries and thus provide a starting infrastructure for capturing avian influenza virus diversity.

## A Cost-effective Surveillance System

Establishing collaborative networks among countries would be cost-effective, reduce the need for additional laboratory capacity in regions of interest for surveillance, and complement and support other surveillance programs in maintaining trained and operational field teams in targeted locations, instead of rebuilding local capacity and logistics for each new avian influenza virus threat. A more robust understanding of virus diversity and changing viral trends might inform biosecurity efforts at the wildlife/domestic animal interface in which virus spillover might occur. This understanding has potential value, given devastation from the death or culling of millions of birds during 1 outbreak of highly pathogenic avian influenza (*26*), because surveillance provides information on genes of low pathogenicity avian influenza viruses that could recombine or mutate to produce high pathogenic avian influenza viruses (*27*).

Stronger capacity for early detection of changing avian influenza virus dynamics in reservoir populations may provide inputs to support public health and pandemic preparedness. These inputs include potentially useful genetic material for vaccine development, detection of virus origins, refined diagnostic capacity beyond influenza A screening, improved understanding of molecular determinants of transmission and pathogenesis from gene segment characterization, and analysis of the potential for virus spread through migration and trade (*28*).

The cost of avian influenza virus outbreaks in poultry is substantial; outbreaks of influenza A(H5N1) virus during 2004–2009 caused US $30 billion in damage (*29*), and the frequency of highly pathogenic avian influenza outbreaks in poultry appears to be increasing (*11*). Rather than sporadically releasing large amounts of funding for wild bird surveillance when specific avian influenza viruses emerge, sustained national, regional, and global investments can provide the targeted baseline level of systematic surveillance we propose. Many countries, especially where avian influenza virus diversity in wild birds is highest, are already investing in some form of avian influenza virus surveillance in wild birds.

Current efforts should be refined by leveling the investment roller coaster that has funded subtype-specific wild bird surveillance toward a lower-cost but long-term investment in collecting and sequencing wild bird avian influenza viruses. Global coordinating bodies, such as OFFLU (a network linking influenza experts and laboratories working with poultry and swine influenzas and World Health Organization expertise) (*13*), provide collaborative forums for government agencies and researchers to compile and share sequences and isolates. For wild bird avian influenza viruses specifically and all influenza viruses, we would be remiss not to work toward coordinated surveillance to support more effective assessment, preparation, and response for emerging influenza viruses that pose potential public health threats.

**Technical Appendix.** Global avian influenza surveillance in wild birds.
