# Supplementary material for: Global Avian Influenza Surveillance in Wild Birds: A Strategy to Capture Viral Diversity
Source: Emerg Infect Dis. 2015 Apr;21(4):e141415. doi: 10.3201/eid2104.141415 (PMC4378471; doi:10.3201/eid2104.141415)
Supplement: Supplementary file 1 — Technical Appendix. Global avian influenza surveillance in wild birds. [file 14-1415-Techapp-s1.pdf]

Article DOI: http://dx.doi.org/10.3201/eid2104.141415

Global Avian Influenza Surveillance in Wild Birds: A Strategy to Capture Viral Diversity

Technical Appendix

Global Avian Influenza Surveillance in Wild Birds

Methods

We analyzed data from 1) a survey of the Organisation for Animal Health (OIE) member countries conducted by OIE, 2) official OIE animal health reports from member countries by using the World Animal Health Information Database (WAHID) interface (http://oie.int/wahid) and the WAHID-Wild interface (http://oie.int/wahidwild); 3) reports of avian influenza wild bird surveillance results published in the peer-reviewed literature by using the Web of Knowledge; and 4) the Influenza Research Database (http://www.fludb.org).

OIE Member Survey

Influenza caused by H7N5 subtype virus that is affecting humans in China is not causing clinical disease in birds. Thus, more proactive approaches to surveillance in birds are necessary to detect viral infection. To determine the scope of current surveillance activities in wild birds, in April 2013, the OIE asked all 178 member countries to provide information on surveillance activities for avian influenza (caused by low pathogenic and highly pathogenic influenza viruses) during the previous 12 months. Results received from 46 of 178 member countries were reviewed.

OIE WAHID Interface Data

We also reviewed avian influenza reporting from the disease timeline database on the WAHID interface (www.oie.int/wahid) during 2008–2012 by using data provided by OIE member countries (data for 2013 were was not available at the time of analysis). Reporting of

avian influenza virus subtypes H5, H7, and highly pathogenic avian influenza virus identified in wild birds to OIE is mandated, and reporting of other subtypes of low pathogenic avian influenza virus is voluntary as part of the OIE Worldwide Monitoring System for Wild Animals. For the purposes of identifying countries reporting surveillance activities for this analysis, we included countries reporting any of the following occurrence categories: confirmed infection but not clinical disease, suspected, confirmed clinical disease, infection/infestation in ≥1 zones, or clinical disease limited to ≥1 zones, disease suspected but not confirmed limited to ≥1 zones, or absent.

Influenza Research Database

To determine other sources of information on avian influenza surveillance in wild birds from outside sources (or potentially in conjunction with) reporting to OIE, we collected wild avian surveillance data from the Influenza Research Database (http://www.fludb.org/) by using a 2-step approach. First, we conducted a search on November 26, 2013, on the Avian Surveillance Summary Search (an option under Search Data→Animal Surveillance) refined for the host type parameter Wild. The search excluded avian surveillance records categorized as captive wild or domestic host type. Total wild bird counts per country were summed. Entries for countries with n = 0 wild bird counts were excluded. By our definition (n>0 wild bird counts per country), reports of avian influenza viruses originating from 40 countries were found. Second, by using the Animal Surveillance Search page, we set display type to customizable summary table. Country was selected as the primary y-axis attribute, and collection year was selected as the primary x-axis attribute. The remaining default search settings were used. The resulting table displayed years with sampling data for each country. For the 40 countries from which there were reports of n>0 wild bird counts, data records were reviewed to determine if wild birds were sampled during January 2008–June 2013 (collection date). Individual collection records were reviewed to ensure that the records reflected n>0 wild birds as opposed to captive wild or domestic birds (noted as behavior classification in the collection record) during the time frame. Most of total effort (sample collection) for countries was reported during 2008–2013.

Web of Knowledge

To explore the extent of reporting on avian influenza surveillance in wild birds in the scientific literature, we searched the Thompson Reuters Web of Knowledge system (www.webofknowledge.com) version 5.12 (all databases) on November 27, 2013, topic search term [Avian Influenza] with publication date parameters for 2008–2013. The search yielded 20,757 results. The search was refined for search term wild bird and yielded 1,220 results. The search was further refined for surveillance, which yielded 431 results. We limited inclusion of reports to those conducting surveillance during 2008–2013. Abstracts were reviewed for surveillance time frames and to confirm n>0 wild bird sampling in the country(s). When results were not clear, full texts were reviewed. Papers needing more review without full-text online versions were searched by using Google Scholar (www.scholar.google.com); if not available on either system, they were not included. Publications focused on surveillance of domestic animals involving experimental infection or investigating surveillance or testing method validation were excluded. In general, information was not included from review papers, except where it was explicit that wild birds had been sampled in a given country within the specified time frame. The list of countries from which reports of conducting wild bird surveillance during January 2008–January 2013 by an article available on Web of Knowledge was compiled (online Appendix Table). Information matching the headings of the OIE survey was also extracted for a subset of 26 countries not responding to the OIE survey but still reporting in the literature available on Web of Knowledge to gain a sense of their sampling, testing, and reporting approaches.

Table. Current and recent surveillance for avian influenza virus in wild birds, by country and income level, reported surveillance by country*

Income level, country	OIE survey	Reporting to OIE	Web of Knowledge	Influenza Research Database
High				
Australia	X	NR	X (*1,2*)	X
Austria	NR	X	X (*3*)	NR
Bahrain	NR	X	NR	NR
Belgium	X	X	NR	NR
Canada	X	X	X (*4,5*)	X
Chile	NR	X	NR	NR
Taiwan	X	X	NR	X
Croatia	NR	X	NR	NR
Czech Republic	X	X	X (*6*)	NR
Denmark	X	X	X (*7*)	NR
Equatorial Guinea	NR	X	NR	NR
Estonia	NR	X	NR	NR
Finland	X	X	NR	NR
France	NR	X	X (*8*)	NR
Germany	NR	X	X (*3,9*)	NR
Guadeloupe	NR	NR	X (*10*)	NR

Income level, country	OIE survey	Reporting to OIE	Web of Knowledge	Influenza Research Database
Hong Kong	NR	NR	X (*11,12*)	NR
Ireland	NR	X	NR	NR
Israel	NR	X	NR	NR
Italy	X	X	X (*13,14*)	NR
Japan	X	X	X (*15–21*)	X
South Korea	X	X	X (*22–28*)	NR
Kuwait	NR	X	NR	NR
Latvia	NR	NR	NR	X
Lithuania	NR	NR	X (*29*)	X
the Netherlands	NR	X	X (*30*)	X
New Caledonia	NR	X	NR	NR
New Zealand	X	X	NR	NR
Norway	X	X	X (*31*)	NR
Oman	NR	NR	NR	X
Poland	X	X	X (*32,33*)	NR
Portugal	X	X	X (*34,35*)	NR
Russia	NR	NR	X (*36–38*)	X
San Marino	NR	X	NR	NR
Saudi Arabia	NR	X	NR	NR
Singapore	X	X	NR	NR
Slovakia	NR	X	X (*39*)	NR
Slovenia	X	X	X (*40*)	NR
Spain	X	X	X (*41*)	NR
Sweden	NR	X	NR	NR
Switzerland	NR	NR	X (*3,42*)	NR
United Kingdom	X	X	X (*43,44*)	NR
United States	X	X	X (*5,44–51*)	X
Uruguay	NR	X	NR	NR
Upper middle				
Algeria	NR	X	NR	NR
Argentina	X	X	X (*52*)	NR
Azerbaijan	NR	X	NR	NR
Bosnia and Herzegovina	X	X	NR	NR
Botswana	NR	X	X (*53,54*)	NR
Brazil	NR	X	NR	NR
Bulgaria	NR	X	X (*55*)	X
China	NR	X	X (*56,57*)	X
Colombia	X	NR	NR	X
Greece	X	NR	NR	X
Greenland	NR	NR	X (*7*)	X
Hungary	X	X	NR	X
Iceland	NR	X	NR	X
Iraq	X	NR	NR	NR
Kazakhstan	X	NR	X (*38*)	NR
Lebanon	NR	NR	NR	X
Libya	NR	X	NR	NR
Malaysia	X	X	NR	NR
Mauritius	X	NR	NR	NR
Mexico	NR	X	X (*5,58*)	NR
Montenegro	NR	X	NR	NR
Namibia	NR	X	NR	NR
Peru	X	X	X (*59*)	NR
Romania	NR	X	NR	X
Serbia	X	X	NR	NR
South Africa	NR	X	X (*53,54*)	NR
Thailand	X	X	X (*60*)	X
Tunisia	X	X	NR	NR
Turkey	X	NR	X (*61*)	X
Lower middle				
Armenia	NR	X	NR	NR
Bhutan	NR	NR	NR	X
Cameroon	NR	NR	NR	X
Côte d'Ivoire	X	X	NR	NR
Egypt	NR	NR	X (*62,63*)	X
El Salvador	NR	X	NR	NR
Georgia	NR	NR	X (*64*)	X
Ghana	NR	NR	X (*65*)	X
Guatemala	NR	NR	X (*66*)	X

Income level, country	OIE survey	Reporting to OIE	Web of Knowledge	Influenza Research Database
India	NR	X	X (*67*)	NR
Lesotho	NR	X	NR	NR
Mauritania	NR	NR	X (*54*)	NR
Mongolia	X	X	X (*21,38,68,69*)	X
Morocco	NR	X	NR	NR
Nigeria	NR	X	X (*70*)	NR
Pakistan	NR	NR	X (*71*)	NR
Papua New Guinea	NR	NR	NR	X
Paraguay	NR	X	NR	NR
Moldova	NR	X	NR	NR
Republic of the Congo	NR	NR	NR	X
Senegal	NR	X	X (*54*)	NR
Sri Lanka	NR	NR	NR	X
Sudan	NR	X	NR	X
Swaziland	NR	X	NR	NR
Ukraine	NR	NR	X (*72*)	NR
Vanuatu	NR	X	NR	NR
Vietnam	X	NR	X (*73,74*)	X
Yemen	X	NR	NR	NR
Zambia	NR	NR	X (*75*)	NR
Low				
Bangladesh	X	X	X (*76*)	X
Benin	NR	X	NR	X
Cambodia	NR	NR	X (*77*)	X
Central African Republic	NR	NR	NR	X
Eritrea	NR	X	NR	NR
Ethiopia	NR	X	NR	NR
Guinea	NR	X	NR	NR
Haiti	NR	X	NR	NR
Kenya	NR	NR	X (*76*)	NR
North Korea	X	NR	NR	NR
Madagascar	NR	X	NR	NR
Mali	NR	NR	X (*54*)	NR
Mozambique	NR	NR	X (*53,54*)	NR
Nepal	NR	X	NR	NR
Tanzania	NR	X	NR	NR
Uganda	NR	X	NR	X
Zimbabwe	NR	X	X (*53,54*)	X

*Reported surveillance by country as per 1) responses to OIE survey confirming surveillance activity; 2) official reports to OIE indicating surveillance activities, 2008–2012; 3) Web of Knowledge literature indicating surveillance activities during 2008–2013; and 4) information reported to the Influenza Research Database. Country income levels are based on the 2013 United Nations categorization. OIE, World Organisation for Animal Health; X, ≥1 report was found in the relevant reporting category; NR, no report. Values in parentheses are references.

References

1. Hansbro PM, Warner S, Tracey JP, Arzey KE, Selleck P, O'Riley K, et al. Surveillance and analysis of avian influenza viruses, Australia. Emerg Infect Dis. 2010;16:1896–904. PubMed http://dx.doi.org/10.3201/eid1612.100776

2. Hoque MA, Burgess GW, Greenhil AR, Hedlefs R, Skerratt LF. Causes of morbidity and mortality of wild aquatic birds at Billabong Sanctuary, Townsville, North Queensland, Australia. Avian Dis. 2012;56:249–56. PubMed http://dx.doi.org/10.1637/9863-072611-Case.1

3. Knight-Jones TJD, Hauser R, Matthes D, Staerk KDC. Evaluation of Effectiveness and Efficiency of Wild Bird Surveillance for Avian Influenza. Vet Res. 2010;41:50. PubMed http://dx.doi.org/10.1051/vetres/2010023

4. Parmley J, Lair S, Leighton FA. Canada's inter-agency wild bird influenza survey. Integrative Zoology. 2009;4:409–17. PubMed http://dx.doi.org/10.1111/j.1749-4877.2009.00177.x

5. Pasick J, Pedersen J, Hernandez MS. Avian influenza in North America, 2009–2011. Avian Dis. 2012;56:845–8. PubMed http://dx.doi.org/10.1637/10206-041512-Reg.1

6. Nagy A, Cernikova L, Krivda V, Hornickova J. Digital genotyping of avian influenza viruses of H7 subtype detected in central Europe in 2007–2011. Virus Res. 2012;165:126–33. PubMed http://dx.doi.org/10.1016/j.virusres.2012.02.005

7. Hulsager CK, Breum SO, Trebbien R, Handberg KJ, Therkildsen OR, Madsen JJ, et al. Surveillance for avian influenza viruses in wild birds in Denmark and Greenland, 2007–10. Avian Dis. 2012;56:992–8. PubMed http://dx.doi.org/10.1637/10190-041012-ResNote.1

8. Lebarbenchon C, Albespy F, Brochet A-L, Grandhomme V, Renaud F, Fritz H, et al. Spread of avian influenza viruses by common teal (*Anas crecca*) in Europe. PLoS ONE. 2009;4:e7289. PubMed http://dx.doi.org/10.1371/journal.pone.0007289

9. Rabl S, Rinder M, Neubauer-Juric A, Bogner K-H, Korbel R, Buettner M. Surveillance of wild birds for avian influenza a virus (AIV) in Bavaria in the years 2007 and 2008 [in German]. Berl Munch Tierarztl Wochenschr. 2009;122:486–93. PubMed

10. Lefrançois T, Hendrikx P, Ehrhardt N, Millien M, Gomez L, Gouyet L, et al. Surveillance of avian influenza in the Caribbean through the Caribbean Animal Health Network: surveillance tools and epidemiologic studies. Avian Dis. 2010;54:369–73. PubMed http://dx.doi.org/10.1637/8787-040109-ResNote.1

11. Leung YH, Cheung P, Zhang L-J, Wu Y-O, Chow K-C, Ho C-K, et al. Influenza viruses in wild birds in Hong Kong, 2003–2010. Influenza Other Respi Viruses. 2011;5:77–8.

12. Smith GJ, Vijaykrishna D, Ellis TM, Dyrting KC, Leung YH, Bahl J, et al. Characterization of avian influenza viruses A (H5N1) from wild birds, Hong Kong, 2004–2008. Emerg Infect Dis. 2009;15:402–7. PubMed http://dx.doi.org/10.3201/eid1503.081190

13. Kelvin AA, Meloni D, Sansonetti P, Borghetto I, Rowe T, Santangelo R, et al. Influenza monitoring in Sardinia, Italy identifies H3 subtype in Mediterranean wild migratory birds. J Infect Dev Ctries. 2012;6:786–97. PubMed http://dx.doi.org/10.3855/jidc.2793

14. Delogu M, Piredda I, Pintore A, Cabras P, Cotti C, Ghetti G, et al. Avian influenza and animal health risk: conservation of endemic threatened wild bbrds in Sardinia Island. Avian Dis. 2012;56:1034–9. PubMed http://dx.doi.org/10.1637/10174-041012-ResNote.1

15. Abao LN, Jamsransuren D, Bui VN, Ngo LH, Trinh DQ, Yamaguchi E, et al. Surveillance and characterization of avian influenza viruses from migratory water birds in eastern Hokkaido, the northern part of Japan, 2009–2010. Virus Genes. 2013;46:323–9. PubMed http://dx.doi.org/10.1007/s11262-012-0868-9

16. Bui VN, Ogawa H, Karibe K, Matsuo K, Nguyen TH, Awad SS, et al. Surveillance of avian influenza virus in migratory water birds in eastern Hokkaido, Japan. J Vet Med Sci. 2011;73:209–15. PubMed http://dx.doi.org/10.1292/jvms.10-0356

17. Fujimoto Y, Ito H, Shivakoti S, Nakamori J, Tsunekuni R, Otsuki K, et al. Avian influenza virus and paramyxovirus isolation from migratory waterfowl and shorebirds in San-in district of western Japan from 2001 to 2008. J Vet Med Sci. 2010;72:963–7. PubMed http://dx.doi.org/10.1292/jvms.10-0012

18. Ito T. Highly pathogenic avian influenza and wild birds. Uirusu. 2009;59:53–8. PubMed http://dx.doi.org/10.2222/jsv.59.53

19. Neagari Y. Challenges for the investigation of causes of wildlife mortality. Japanese Journal of Zoo and Wildlife Medicine. 2013;18:53–9.

20. Samad RA, Sakoda Y, Tsuda Y, Simulundu E, Manzoor R, Okamatsu M, et al. Virological surveillance and phylogenetic analysis of the PB2 genes of influenza viruses isolated from wild water birds flying from their nesting lakes in Siberia to Hokkaido, Japan in autumn. Jpn J Vet Res. 2011;59:15–22. PubMed

21. Yamamoto N, Sakoda Y, Motoshima M, Yoshino F, Soda K, Okamatsu M, et al. Characterization of a non-pathogenic H5N1 influenza virus isolated from a migratory duck flying from Siberia in Hokkaido, Japan, in October 2009. Virol J. 2011;8:65. PubMed http://dx.doi.org/10.1186/1743-422X-8-65

22. Baek YH, Pascua PN, Song M-S, Park KJ, Kwon HI, Lee JH, et al. Surveillance and characterization of low pathogenic H5 avian influenza viruses isolated from wild migratory birds in Korea. Virus Res. 2010;150:119–28. PubMed http://dx.doi.org/10.1016/j.virusres.2010.03.002

23. Choi JG, Kang HM, Jeon WJ, Choi KS, Kim KI, Song BM, et al. Characterization of clade 2.3.2.1 H5N1 highly pathogenic avian influenza viruses isolated from wild birds (mandarin duck and

Eurasian eagle owl) in 2010 in Korea. Viruses. 2013;5:1153–74. PubMed http://dx.doi.org/10.3390/v5041153

24. Kang H-M, Choi J-G, Kim K-I, Park H-Y, Park C-K, Lee Y-J. Genetic and antigenic characteristics of H4 subtype avian influenza viruses in Korea and their pathogenicity in quails, domestic ducks and mice. J Gen Virol. 2013;94:30–9. PubMed http://dx.doi.org/10.1099/vir.0.046581-0

25. Kang HM, Jeong OM, Kim MC, Kwon JS, Paek MR, Choi JG, et al. Surveillance of avian influenza virus in wild bird fecal samples from South Korea, 2003–2008. J Wildl Dis. 2010;46:878–88. PubMed http://dx.doi.org/10.7589/0090-3558-46.3.878

26. Kim H-R, Park C-K, Lee Y-J, Oem J-K, Kang H-M, Choi J-G, et al. Low pathogenic H7 subtype avian influenza viruses isolated from domestic ducks in South Korea and the close association with isolates of wild birds. J Gen Virol. 2012;93:1278–87. PubMed http://dx.doi.org/10.1099/vir.0.041269-0

27. Kwon HI, Song M-S, Pascua PN, Baek YH, Lee JH, Hong S-P, et al. Genetic characterization and pathogenicity assessment of highly pathogenic H5N1 avian influenza viruses isolated from migratory wild birds in 2011, South Korea. Virus Res. 2011;160:305–15. PubMed http://dx.doi.org/10.1016/j.virusres.2011.07.003

28. Lee D-H, Park J-K, Youn H-N, Lee Y-N, Lim T-H, Kim M-S, et al. Surveillance and isolation of HPAI H5N1 from wild mandarin ducks (*Aix galericulata*). J Wildl Dis. 2011;47:994–8. PubMed http://dx.doi.org/10.7589/0090-3558-47.4.994

29. Svazas S, Morkunas M, Verhagen J, Fouchier R, Morkunas J, Mundkur T, et al. Surveillance of wild waterbirds for avian influenza viruses in Lithuania. Veterinarija Ir Zootechika. 2012;60:72–8.

30. Verhagen JH, Munster VJ, Majoor F, Lexmond P, Oanh V, Stumpel JBG, et al. Avian influenza A virus in wild birds in highly urbanized areas. PLoS ONE. 2012;7:e38256. PubMed http://dx.doi.org/10.1371/journal.pone.0038256

31. Tønnessen R, Kristoffersen AB, Jonassen CM, Hjortaas MJ, Hansen EF, Rimstad E, et al. Molecular and epidemiological characterization of avian influenza viruses from gulls and dabbling ducks in Norway. Virol J. 2013;:10:112. PubMed http://dx.doi.org/10.1186/1743-422X-10-112

32. Smietanka K, Minta Z, Wlodarczyk R, Wyrostek K, Jozwiak M, Olszewska M, et al. Avian influenza viruses in wild birds at the Jeziorsko reservoir in Poland in 2008–2010. Pol J Vet Sci. 2012;15:323–8. PubMed http://dx.doi.org/10.2478/v10181-011-0150-4

33. Smietanka K, Pikula A, Minta Z, Meissner W. Evidence of persistence and multiple genetic modifications of H7N7 low-pathogenic avian influenza virus in wild mallards in Poland provided by phylogenetic studies. Avian Pathol. 2011;40:131–8. PubMed http://dx.doi.org/10.1080/03079457.2010.537304

34. Tolf C, Bengtsson D, Rodrigues D, Latorre-Margalef N, Wille M, Figueiredo ME, et al. Birds and viruses at a crossroad: surveillance of influenza A virus in Portuguese waterfowl. PLoS ONE. 2012;7:e49002. PubMed http://dx.doi.org/10.1371/journal.pone.0049002

35. Henriques AM, Fagulha T, Barros SC, Ramos F, Duarte M, Luis T, et al. Multiyear surveillance of influenza A virus in wild birds in Portugal. Avian Pathol. 2011;40:597–602. PubMed http://dx.doi.org/10.1080/03079457.2011.618943

36. Sivay MV, Baranovich T, Marchenko VY, Sharshov KA, Govorkova EA, Shestopalov AM, et al. Influenza A (H15N4) virus isolation in western Siberia, Russia. J Virol. 2013;87:3578–82. PubMed http://dx.doi.org/10.1128/JVI.02521-12

37. Sivay MV, Sayfutdinova SG, Sharshov KA, Alekseev AY, Yurlov AK, Runstadler J, et al. Surveillance of influenza A virus in wild birds in the Asian portion of Russia in 2008. Avian Dis. 2012;56:456–63. PubMed http://dx.doi.org/10.1637/9868-080111-Reg.1

38. Marchenko VY, Alekseev AY, Sharshov KA, Petrov VN, Silko NY, Susloparov IM, et al. Ecology of influenza virus in wild bird populations in central Asia. Avian Dis. 2012;56:234–7. PubMed http://dx.doi.org/10.1637/9834-061611-ResNote.1

39. Borovska P, Kabat P, Ficova M, Trnka A, Svetlikova D, Betakova T. Prevalence of avian influenza viruses, *Mycobacterium avium*, and *Mycobacterium avium* subsp *paratuberculosis* in marsh-dwelling passerines in Slovakia, 2008. Biologia. 2011;66:282–7. http://dx.doi.org/10.2478/s11756-011-0016-3

40. Slavec B, Krapez U, Racnik J, Hari A, Wernig JM, Dovc A, et al. Surveillance of influenza A viruses in wild birds in Slovenia from 2006 to 2010. Avian Dis. 2012;56:999–1005. PubMed http://dx.doi.org/10.1637/10175-041012-ResNote.1

41. Pérez-Ramirez E, Acevedo P, Allepuz A, Gerrikagoitia X, Alba A, Busquets N, et al. Ecological factors driving avian influenza virus dynamics in Spanish wetland ecosystems. PLoS ONE. 2012;7:e46418. PubMed http://dx.doi.org/10.1371/journal.pone.0046418

42. Baumer A, Feldmann J, Renzullo S, Mueller M, Thuer B, Hofmann MA. Epidemiology of avian influenza virus in wild birds in Switzerland between 2006 and 2009. Avian Dis. 2010;54:875–84. PubMed http://dx.doi.org/10.1637/9119-110209-Reg.1

43. Breed AC, Irvine RM, Duncan D, Rae D, Snow L, Cook AJC, et al. An evaluation of wild bird avian influenza surveillance in Great Britain. Avian Dis. 2012;56:986–91. PubMed http://dx.doi.org/10.1637/10166-040912-Reg.1

44. Siembieda JL, Johnson CK, Cardona C, Anchell N, Dao N, Reisen W, et al. Influenza A viruses in wild birds of the Pacific Flyway, 2005–2008. Vector Borne Zoonotic Dis. 2010;10:793–800. PubMed http://dx.doi.org/10.1089/vbz.2009.0095

45. Kistler WM, Stallknecht DE, Deliberto TJ, Swafford S, Pedersen K, Van Why K, et al. Antibodies to avian influenza viruses in Canada geese (*Branta canadensis*): a potential surveillance tool? J Wildl Dis. 2012;48:1097–101. PubMed http://dx.doi.org/10.7589/2011-02-046

46. Hill NJ, Takekawa JY, Cardona CJ, Meixell BW, Ackerman JT, Runstadler JA, et al. Cross-seasonal patterns of avian influenza virus in breeding and wintering migratory birds: a fyway perspective. Vector Borne Zoonotic Dis. 2012;12:243–53. PubMed http://dx.doi.org/10.1089/vbz.2010.0246

47. Ferro PJ, Khan O, Peterson MJ, Batchuluun D, Reddy SM, Lupiani B. Avian influenza virus surveillance in hunter-harvested waterfowl, Texas Coast, September 2009–January 2010. Avian Dis. 2012;56:1006–9. PubMed http://dx.doi.org/10.1637/10194-041012-ResNote.1

48. Reeves AB, Pearce JM, Ramey AM, Ely CR, Schmutz JA, Flint PL, et al. Genomic analysis of avian influenza viruses from waterfowl in western Alaska, USA. J Wildl Dis. 2013;49:600–10. PubMed http://dx.doi.org/10.7589/2012-04-108

49. Ely CR, Hall JS, Schmutz JA, Pearce JM, Terenzi J, Sedinger JS, et al. Evidence that life history characteristics of wild birds influence infection and exposure to influenza A viruses. PLoS ONE. 2013;8:e57614. PubMed http://dx.doi.org/10.1371/journal.pone.0057614

50. Pedersen K, Swafford SR, DeLiberto TJ. Low pathogenicity avian influenza subtypes isolated from wild birds in the United States, 2006–2008. Avian Dis. 2010;54:405–10. PubMed http://dx.doi.org/10.1637/8693-031309-Reg.1

51. Hildebrand E, Carstensen M, Butler E. Surveillance for highly pathogenic avian influenza in Minnesota’s migratory birds from 2006–2010. Summaries of wildlife research findings. St. Paul MN): Minnesota Department of Natural Resources; 2010. p. 203–11.

52. Buscaglia C. A survey for avian influenza from gulls on the coasts of the District of Pinamar and the Lagoon Salada Grande, General Madariaga, Argentina. Avian Dis. 2012;56:1017–20. PubMed http://dx.doi.org/10.1637/10147-040712-Reg.1

53. Cumming GS, Caron A, Abolnik C, Cattoli G, Bruinzeel LW, Burger CE, et al. The ecology of influenza A viruses in wild birds in southern Africa. EcoHealth. 2011;8:4–13. PubMed http://dx.doi.org/10.1007/s10393-011-0684-z

54. Gaidet N, Caron A, Cappelle J, Cumming GS, Balanca G, Hammoumi S, et al. Understanding the ecological drivers of avian influenza virus infection in wildfowl: a continental-scale study across Africa. Proc Biol Sci. 2012;279:1131–41. PubMed http://dx.doi.org/10.1098/rspb.2011.1417

55. Goujgoulova G, Petkova AM, Georgiev G. Avian influenza viruses isolated from mallards in Bulgaria. Avian Dis. 2010;54:450–2. PubMed http://dx.doi.org/10.1637/8681-030709-ResNote.1

56. Han Y, Hou G, Jiang W, Han C, Liu S, Chen J, et al. A survey of avian influenza in tree sparrows in China in 2011. PLoS ONE. 2012;7:e33092. PubMed http://dx.doi.org/10.1371/journal.pone.0033092

57. Zhu G, Wang RJ, Xuan FJ, Daszak P, Anthony SJ, Zhang SY, et al. Characterization of recombinant H9N2 influenza viruses isolated from wild ducks in China. Vet Microbiol. 2013;166:327–36. PubMed http://dx.doi.org/10.1016/j.vetmic.2013.05.013

58. Montalvo-Corral M, Lopez-Robles G, Hernandez J. Avian influenza survey in migrating waterfowl in Sonora, Mexico. Transbound Emerg Dis. 2011;58:63–8. PubMed http://dx.doi.org/10.1111/j.1865-1682.2010.01182.x

59. Williams RA, Segovia-Hinostroza K, Ghersi BM, Gonzaga V, Peterson AT, Montgomery JM. Avian influenza infections in nonmigrant land birds in Andean Peru. J Wildl Dis. 2012;48:910–7. PubMed http://dx.doi.org/10.7589/2011-02-052

60. Siengsanan-Lamont J, Robertson I, Blacksell SD, Ellis T, Fenwick S, Saengchoowong S, et al. Virological and molecular epidemiological investigations into the role of wild birds in the epidemiology of influenza A/H5N1 in central Thailand. Vet Microbiol. 2011;148:213–8. PubMed http://dx.doi.org/10.1016/j.vetmic.2010.09.028

61. Boynukara B, Gulhan T, Adizel O, Ilhan Z, Aksakal A, Durmus A, et al. Determination of avian influenza A viruses in some avian species in Van Lake Basin by real-time PCR, isolation and subtyping. Journal of Animal and Veterinary Advances. 2011;10:502–10. http://dx.doi.org/10.3923/javaa.2011.502.510

62. El-Zoghby EF, Aly MM, Nasef SA, Hassan MK, Arafa AS, Selim AA, et al. Surveillance on A/H5N1 virus in domestic poultry and wild birds in Egypt. Virol J. 2013;10:203. PubMed http://dx.doi.org/10.1186/1743-422X-10-203

63. Soliman A, Saad M, Elassal E, Amir E, Plathonoff C, Bahgat V, et al. Surveillance of avian influenza viruses in migratory birds in Egypt, 2003–09. J Wildl Dis. 2012;48:669–75. PubMed http://dx.doi.org/10.7589/0090-3558-48.3.669

64. Lewis NS, Javakhishvili Z, Russell CA, Machablishvili A, Lexmond P, Verhagen JH, et al. Avian influenza virus surveillance in wild birds in Georgia: 2009–2011. PLoS ONE. 2013;8:e58534. PubMed http://dx.doi.org/10.1371/journal.pone.0058534

65. Odoom JK, Bel-Nono S, Rodgers D, Agbenohevi PG, Dafeamekpor CK, Sowa RM, et al. Troop education and avian influenza surveillance in military barracks in Ghana, 2011. BMC Public Health. 2012;12:957. PubMed http://dx.doi.org/10.1186/1471-2458-12-957

66. González-Reiche AS, Morales-Betoulle ME, Alvarez D, Betoulle J-L, Mueller ML, Sosa SM, et al. Influenza A viruses from wild birds in Guatemala belong to the North American lineage. PLoS ONE. 2012;7:e32873. PubMed http://dx.doi.org/10.1371/journal.pone.0032873

67. Pawar SD, Kale SD, Rawankar AS, Koratkar SS, Raut CG, Pande SA, et al. Avian influenza surveillance reveals presence of low pathogenic avian influenza viruses in poultry during 2009–2011 in the West Bengal State, India. Virol J. 2012;9:151. PubMed http://dx.doi.org/10.1186/1743-422X-9-151

68. Gilbert M, Jambal L, Karesh WB, Fine A, Shiilegdamba E, Dulam P, et al. Highly pathogenic avian influenza virus among wild birds in Mongolia. PLoS ONE. 2012;7:e44097. PubMed http://dx.doi.org/10.1371/journal.pone.0044097

69. Sakoda Y, Sugar S, Batchluun D, Erdene-Ochir T-O, Okamatsu M, Isoda N, et al. Characterization of H5N1 highly pathogenic avian influenza virus strains isolated from migratory waterfowl in Mongolia on the way back from southern Asia to their northern territory. Virology. 2010;406:88–94. PubMed http://dx.doi.org/10.1016/j.virol.2010.07.007

70. Columba Teru V, Manu SA, Ahmed GI, Junaidu K, Newman S, Nyager J, et al. Situation-based survey of avian influenza viruses in possible “bridge” species of wild and domestic birds in Nigeria. Influenza Res Treat. 2012;2012:8567601. Epub 2012 Sep 2. doi: 10.1155/2012/567601.

71. Siddique N, Naeem K, Abbas MA, Ahmed Z, Malik SA. Sequence and phylogenetic analysis of highly pathogenic avian influenza H5N1 viruses isolated during 2006–2008 outbreaks in Pakistan

reveals genetic diversity. Virol J. 2012;9:300–13. PubMed http://dx.doi.org/10.1186/1743-422X-9-300

72. Kulak MV, Ilinykh FA, Zaykovskaya AV, Epanchinzeva AV, Evstaphiev IL, Tovtunec NN, et al. Surveillance and identification of influenza A viruses in wild aquatic birds in the Crimea, Ukraine (2006–2008). Avian Dis. 2010;54:1086–90. PubMed http://dx.doi.org/10.1637/9272-020510-ResNote.1

73. Takakuwa H, Yamashiro T, Le MQ, Phuong LS, Ozaki H, Tsunekuni R, et al. The characterization low pathogenic avian influenza viruses isolated from wild birds in northern Vietnam from 2006 to 20090. Comp Immunol Microbiol Infect Dis. 2013;36:581–90. PubMed http://dx.doi.org/10.1016/j.cimid.2013.06.004

74. Thinh TV, Gilbert M, Bunpapong N, Amonsin A, Nguyen DT, Doherty PF Jr, et al. Avian influenza viruses in wild land birds in northern Vietnam. J Wildl Dis. 2012;48:195–200. PubMed http://dx.doi.org/10.7589/0090-3558-48.1.195

75. Simulundu E, Ishii A, Igarashi M, Mweene AS, Suzuki Y, Hang'ombe BM, et al. Characterization of influenza A viruses isolated from wild waterfowl in Zambia. J Gen Virol. 2011;92:1416–27. PubMed http://dx.doi.org/10.1099/vir.0.030403-0

76. Khatun A, Giasuddin M, Islam KM, Khanom S, Samad MA, Islam MR, et al. Surveillance of avian influenza virus type A in semi-scavenging ducks in Bangladesh. BMC Vet Res. 2013;9:196. PubMed http://dx.doi.org/10.1186/1746-6148-9-196

77. Theary R, San S, Davun H, Allal L, Lu HG. New outbreaks of H5N1 highly pathogenic avian influenza in domestic poultry and wild birds in Cambodia in 2011. Avian Dis. 2012;56:861–4. PubMed http://dx.doi.org/10.1637/10195-041012-ResNote.1

78. Ofula VO, Franklin AB, Root JJ, Sullivan HJ, Gichuki P, Makio A, et al. Detection of avian influenza viruses in wild waterbirds in the Rift Valley of Kenya using fecal sampling. Vector Borne Zoonotic Dis. 2013;13:394–400. PubMed http://dx.doi.org/10.1089/vbz.2011.0926
